# Experiences, challenges and lessons while implementing a clinical decision support system in Botswana

**DOI:** 10.1093/oodh/oqaf014

**Published:** 2025-10-01

**Authors:** Kagiso Ndlovu, Nate Stein, Ruth Gaopelo, Mosadikhumo Monkge, Laura Moen, Mmoloki C Molwantwa

**Affiliations:** Department of Health Informatics, Sir Ketumile Masire Teaching Hospital, Unit 12, Sir Ketumile Masire Teaching Hospital, P.O Box 423 AAD, Poso House, 00267 Gaborone, Botswana; Department of Product Management, VisualDx, 20 Audubon St., Rochester, NY 14610, USA; Department of Computer Science, University of Botswana, Notwane Road, Plot 4775, Private Bag UB 0022, Gaborone, 00267, Botswana; Department of Paediatrics and Adolescent Health, Princess Marina Hospital, Plot 1836, North Ring Road, Extension 11, Private Bag BO 320, Gaborone, Botswana; Department of Life Sciences, VisualDx, 1009 Wade Avenue, Raleigh, NC 27605, USA; Department of Medical Education, University of Botswana, Notwane Road, Plot 4775, Private Bag UB 0022, Gaborone, 00267, Botswana

**Keywords:** VisualDx, dermatology, mHealth, clinical decision support system, Botswana

## Abstract

The use of information and communication technologies in healthcare has given rise to mobile health applications and services. For the developing world, mobile health has been hailed as being valuable for extending access to healthcare to underserved populations. More recently, mobile health applications support clinicians to quickly navigate decision making processes. An exemplar decision support system, VisualDx, was implemented in Botswana to provide reference materials at the point of care to support early diagnosis and management of complex dermatological conditions. This study shares experiences, challenges and lessons learnt while implementing VisualDx in Botswana. An explanatory sequential mixed methods feasibility study was conducted with 28 healthcare providers stationed at 20 clinics and hospital sites across Botswana. Nine recorded training sessions were conducted via Zoom and participants thereafter interacted with VisualDx on varying use cases. Quantitative and qualitative data were collected via surveys and a semi-structured interview per participant. Standard VisualDx App usage data was also collected. Descriptive statistics were generated and analyzed. Thematic analysis of interview transcripts was performed using Delve. Experiences, challenges and lessons learned throughout VisualDx implementation in Botswana cut across; Infrastructure, Data protection compliance, Image data quality, Continuous training support, artificial intelligence regulation, Participants’ retention and Sustainable digital health funding. The implementation of VisualDx in Botswana illustrates both the value and the challenges of cross-sector and cross-border collaboration in driving adoption of eHealth tools. The lessons learned may inform future strategy for implementation of other eHealth platforms in Botswana and other similar developing countries.

## BACKGROUND

Globally, health systems are increasingly burdened by the rising costs of healthcare provision and persistent challenges related to inadequate human resources for health, particularly in low- and middle-income countries (LMICs) [1]. These challenges present complex health problems that demand innovative and sustainable solutions. One such solution is the cost-effective application of information and communication technologies (ICTs) in healthcare—commonly referred to as eHealth [2]. eHealth is widely recognized as a critical component in addressing both current and emerging healthcare challenges.

The World Health Organization (WHO) underscores the essential role of eHealth, identifying it as a key enabler in achieving Universal Health Coverage (UHC) and health-related Sustainable Development Goals (SDGs) [3]. This perspective is particularly relevant within the African context, where the healthcare burden is substantial and resources are often limited [4]. In response, many governments are increasingly turning to data-driven interventions such as Clinical Decision Support Systems (CDSS), which have the potential to augment human decision-making and enhance clinical efficiency [5].

Botswana serves as a representative example of an LMIC actively engaging with these innovations. The country recently launched its National eHealth Strategy (2020–2024), which positions eHealth as a strategic tool for improving healthcare delivery and outcomes [6]. The strategy highlights the value of emerging technologies, including the use of mobile devices and the Internet of Things (IoT), with specific emphasis on the potential of sensor technologies to populate digital systems with real-time health data (Section 2.2.3) [6].

Although less severe than in other parts of Sub-Saharan Africa (SSA), Botswana faces a well-documented shortage of health human resources (HHR), particularly within the primary healthcare sector [7]. The country has a low doctor-to-patient ratio of 38 per 10 000 population, which falls below the World Health Organization’s recommended ratio of 45 per 10 000 [8]. This shortfall is compounded by a scarcity of medical specialists, which remains a significant barrier to achieving high-quality healthcare in Botswana [8].

Nkomazana *et al*. [7] identify several contributing factors to this shortage, including high staff turnover across all levels of the health sector, inequitable deployment of personnel and inadequate optimization of the existing workforce. The dermatology sub-speciality exemplifies this challenge: Botswana’s public health system has historically relied on a limited number of dermatologists, with past staffing levels ranging from no permanent specialists to two full-time Ministry of Health (MOH) employees and three contract dermatologists from Cuba [9].

At the time of writing, there were reportedly four government-employed dermatologists stationed in Molepolole, Francistown, Gaborone and Maun, respectively. However, the demand for dermatological care significantly exceeds the available specialist capacity, often resulting in patient wait times of 6 months or more for consultations [9]. Consequently, the management of dermatological conditions is frequently delegated to general practitioners or nurses, many of whom serve as the primary healthcare providers in rural and underserved areas [10].

This pronounced shortage highlights the urgent need for more efficient use of limited specialist resources and the ongoing empowerment of non-specialist healthcare workers involved in skin disease management [11]. It also underscores the potential value of implementing CDSS as a means of addressing these challenges and enhancing the quality of dermatological care delivery in Botswana.

In 2021, the University of Botswana (UB) partnered with VisualDx on a research study funded by the Bill & Melinda Gates Foundation (Grant No. INV003773). The aim of the study was to assess the feasibility of implementing VisualDx in patient care settings across Botswana and to collect user feedback to guide further development of the platform [12]. This current study builds upon a previous study conducted by the authors [12].

VisualDx is a CDSS with over two decades of experience in aiding healthcare professionals during the clinical decision-making process [13]. Developed by VisualDx, the platform is designed for use on mobile devices operating on Android (Google, Inc., Mountain View, CA, USA) and iOS (Apple Inc., USA), and it can also be accessed via web browsers on desktop computers. As part of the Botswana study, VisualDx developed an offline version of its application specifically for Android devices, further increasing its accessibility in low-connectivity environments. The company employs ˃70 full-time staff, all focused on ensuring the platform delivers accurate, current content with user-friendly functionality. To date, VisualDx has been adopted by over 2300 universities, hospitals and clinical institutions worldwide [13].

VisualDx integrates expert-curated clinical knowledge, problem-oriented search functionality, an extensive medical image library and advanced technologies to support differential diagnosis, treatment planning and patient education. The platform has demonstrated potential to enhance provider confidence and reduce diagnostic errors, particularly in primary care contexts [14, 15]. A key feature of the platform, DermExpert™, leverages a Convolutional Neural Network (CNN) to analyze clinical images and provide diagnostic suggestions. CNNs are a type of machine learning model trained on large datasets of labeled images and clinical findings, enabling them to recognize patterns and support diagnostic accuracy in dermatology and other visual specialties [16].

While CDSS offer several well-documented benefits, it is equally important to consider the challenges associated with their implementation. This study explores the experiences, challenges and key lessons learned during the deployment of the VisualDx CDSS in clinical settings across Botswana.

## METHODS

### Study participants

The implementation of the VisualDx platform in Botswana was supported by healthcare workers based in dermatology clinics across both public and private health facilities, as well as medical students from the University of Botswana who were enrolled in dermatology coursework or clinical rotations. Prospective participants were recruited through email and WhatsApp invitations disseminated by the eHealth Research Unit at the University of Botswana (UB). A total of 18 individuals initially volunteered to participate in the study and were subsequently provided with informed consent forms via email.

Following the easing of COVID-19 restrictions in Botswana, an additional 10 healthcare workers were recruited through the Greater Gaborone District Health Management Team (DHMT) to join the study, with ⁓3 months remaining in the data collection period. This brought the total number of participants to 28, representing 20 sites, including healthcare facilities and the University of Botswana. The DHMT, a local authority operating under the Ministry of Health (MOH), is responsible for overseeing the management and staffing of primary care clinics. Participants were distributed across six health districts: Greater Gaborone (21), Greater Palapye (1), Greater Phikwe (2), Greater Francistown (2), Maun (1) and Chobe (1).

The authors acknowledge the study’s limited sample size and attribute these limitations in part to funding constraints, which restricted the ability to provide mobile devices to participants. This, in turn, affected participation rates, as not all individuals were willing or able to use their personal mobile phones for the duration of the project. Financial limitations also curtailed the scale and reach of recruitment efforts. Additionally, the COVID-19 pandemic placed significant strain on the healthcare system in Botswana, further impacting participation. Many healthcare providers faced uncertainty regarding their postings, workload and availability, which affected their willingness or capacity to engage in the study. Consequently, the sample was biased toward participants who were already inclined to use mobile health (mHealth) tools in their routine clinical practice.

### VisualDx use

All participants accessed the VisualDx mobile application using their personal smartphones or tablet devices, with login credentials provided by VisualDx. To support application installation and ongoing usage, participants were provided with mobile data vouchers to offset associated costs. Initial training for the first cohort was delivered via the Zoom platform at the time of enrolment, while participants recruited through the District Health Management Team (DHMT) attended an in-person training session hosted by the University of Botswana’s eHealth Research Unit.

The training sessions covered foundational information technology skills, demonstrations of key VisualDx features and guidance on applying the platform to common dermatological and general medical conditions encountered in Botswana. Recordings of the training were made available to those unable to attend in real time. Over the course of the study, six case-based training sessions were conducted to illustrate the effective use of VisualDx in supporting clinical reasoning. Participants were encouraged to use the platform at their discretion throughout the study period. Additionally, a WhatsApp group was established to facilitate communication, disseminate announcements and provide ongoing technical and peer support.

### Data collection

An explanatory sequential mixed methods design was employed to evaluate the feasibility and acceptability of VisualDx as a Clinical Decision Support System (CDSS) in Botswana [17]. This design was selected to enable the researchers to quantitatively assess participants’ acceptance of the CDSS and subsequently explore, through qualitative inquiry, the underlying reasons for their survey responses. This approach allowed for a more comprehensive understanding of participants’ experiences, challenges and lessons learned during the implementation of the tool.

Quantitative data were collected through three surveys administered at the beginning, midpoint and end of the study period. This longitudinal design was intended to capture changes in participants’ acceptance and use of the system as their familiarity with it evolved. The initial draft of the survey instruments and interview script was developed by RG and NS, and subsequently reviewed and refined by all authors to eliminate ambiguities and improve clarity. Once finalized, the surveys were programmed into the Research Electronic Data Capture (REDCap) system (Vanderbilt University) [18]. Pre-testing of the survey instruments was conducted by RG, NS and KN, and the surveys were further optimized using enhanced branching logic to improve the user experience.

All surveys were administered through the REDCap, with links provided to the participants for access on their personal or work devices. REDCap is a secure (HIPAA and GDPR compliant) system for supporting electronic data capture for research and operational support projects. The first survey was distributed to participants immediately following their initial mobile application training (March 2021). The second survey was delivered in the third month of the study period (May 2021). For the cohort of participants that started midway through the study, this survey was delivered after 1 month of participation (July 2021). The final survey was completed at the end of the study period (August 2021). All three surveys were offered to the same participants, but due to their conflicting work schedules not all were able to participate. Surveys had closed-ended questions (dichotomous, multiple choice and Likert scale) and open-ended questions.

Starting 3 months into the study period in June 2021, participants were contacted individually via WhatsApp to schedule semi-structured interviews. All interviews were conducted remotely via Zoom platform, with each interview recorded, transcribed verbatim and reviewed by all researchers. Qualitative data from semi-structured interviews was collected (one 30–60 min interview with each participant) 1–3 months after each participant’s completion of the initial study survey, to further gain in depth insights from participants. Both survey and interview tools were used by the authors to meet the study objectives.

VisualDx mobile application usage data was also collected from the VisualDx servers through existing event tracking mechanisms.

Structured Query Language (SQL) statements were executed on the VisualDx database to extract usage data linked to the user accounts of study participants.

### Data analysis

Quantitative data were summarized using descriptive statistics, with calculations of mean, median, range and standard deviation performed within the REDCap system. For qualitative analysis, interview transcripts were uploaded into Delve software for systematic coding. Thematic analysis was conducted following Braun and Clarke’s widely recognized framework [19], allowing for the identification and organization of key themes. An iterative approach to transcript review and deductive coding [20] was independently carried out by NS, RG and MM to ensure rigor and consistency in the analytical process.

Usage data linked to study participants’ user accounts were analyzed using Microsoft Excel to generate basic descriptive statistics, focusing on frequency of use, mode of access (e.g. mobile or web) and the features most frequently utilized.

Integration of the mixed methods was accomplished through a weaving narrative approach, which connected emerging themes from the qualitative interviews with findings from the quantitative survey data to provide a cohesive interpretation of the study results.

### Ethics approval

The study protocol received ethical approval from the University of Botswana’s Institutional Review Board (IRB) (Reference: UBR/RES/IRB/BIO/223) and the Botswana Ministry of Health (MOH) (Reference: HPDME: 13/18/1) in December 2020. Following approval, the protocol was implemented over a 6-month period, from March to August 2021.

## RESULTS

Study participants consisted of healthcare workers at public and private health facilities as well as medical students from the University of Botswana participating in dermatology coursework or rotations at health facilities (Table 1).

**Table 1:** Study participants characteristics

Participants Specialty	Age range	N	Site
Obstetrician and gynecologist	30–39	1	Nyangabwe Hospital
Dermatologist	40–49	2	Princess Marina Hospital Nyangabwe Hospital
General Practice/Nurse	20–29	8	Hospital way Medical Center Mahalapye DHMT Shakawe Clinic Princess Marina
General Practice/Nurse	30–39 40–49	10 1	Princess Marina Hospital Otse Clinic Old Naledi Clinic Siga Clinic Baylor Children’s Hospital Gaborone West Clinic Nkoyaphiri Clinic Block 8 Clinic
Medical Student	20–29	1	University of Botswana/Princess Marina Hospital
Midwifery	40–49	1	Selibe Phikwe Hospital
Family Medicine	30–39 20–29	1 1	Maun Hospital Princess Marina Hospital
Surgery	20–29	1	Princess Marina Hospital
Public Health	30–39	1	University of Botswana

**Figure 1:**
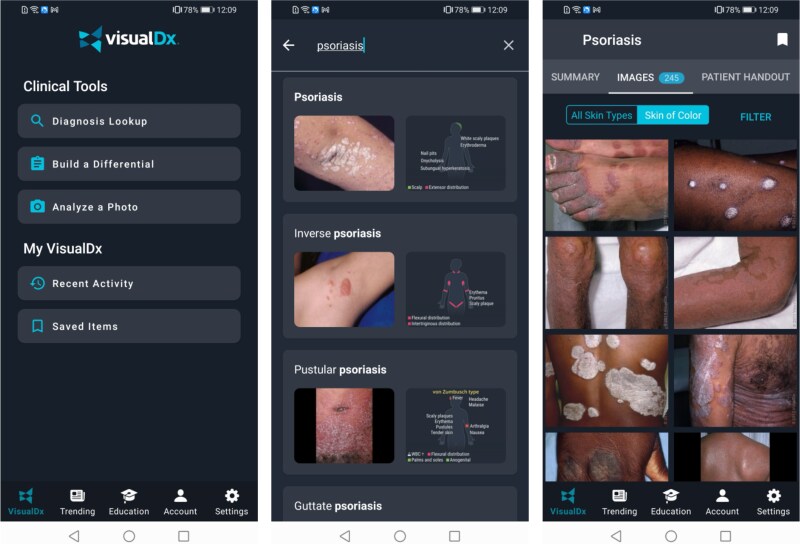
Searching for a diagnosis to view images and detailed diagnosis information

**Figure 2:**
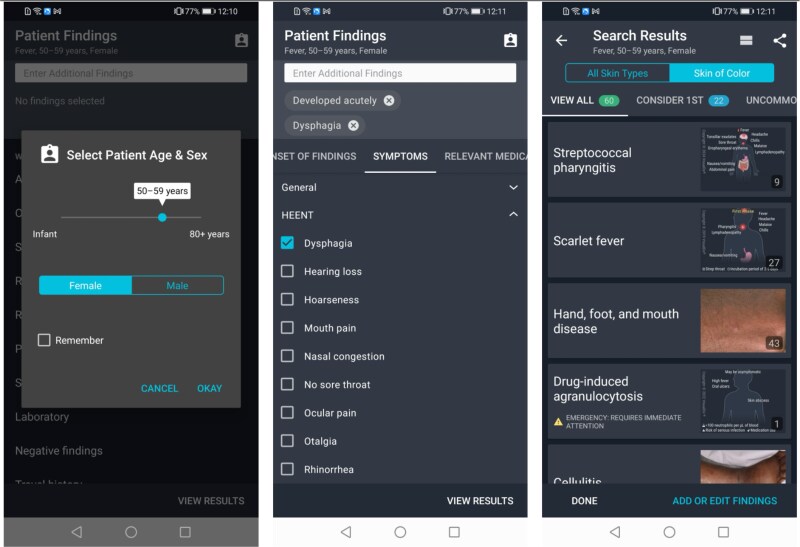
Building a differential diagnosis based on the patient’s symptoms

**Figure 3:**
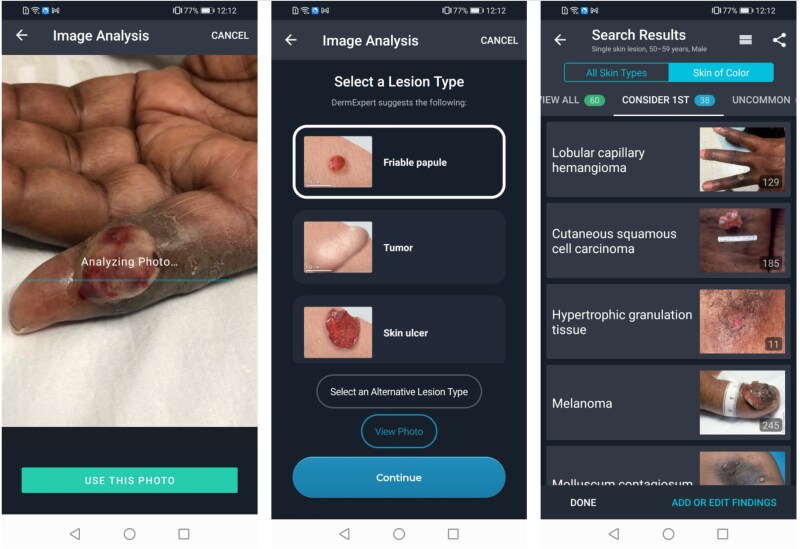
Using VisualDx’s DermExpert™ feature to analyze a skin problem with artificial intelligence

**Figure 4:**
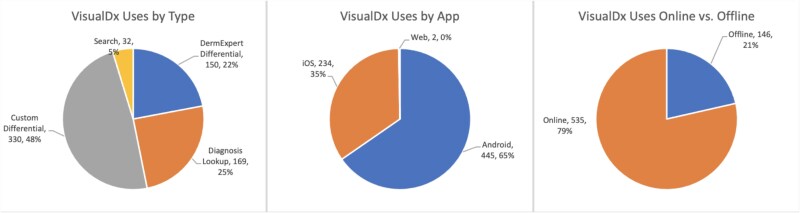
VisualDx usage summary by use case, by operating system and by connectivity mode

**Figure 5:**
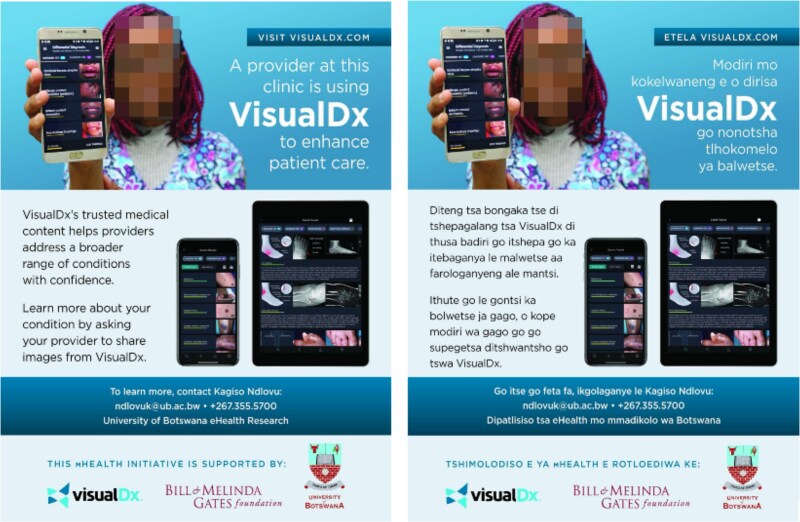
VisualDx sensitization posters at clinics (English and Setswana versions)

This paper reports on experiences (Tables 2 and 3), challenges and resolutions while implementing VisualDx in Botswana (Table 4). Figures 1–5 are intended to illustrate specific implementation features of the VisualDx platform and how information on VisualDx was shared through posters at health facilities in Botswana.

**Table 2:** Rating of mid-pilot participants’ experiences

Mid Pilot Experiences (22 respondents)	Yes	No	Unsure
Have you encountered any issues or barriers when trying to use VisualDx?	7 (32%)	15 (68%)	0 (0%)
Have you encountered any scenarios where using VisualDx provided a clear benefit to you or your patient?	19 (86%)	3 (14%)	0 (0%)
Do you feel that the information you gain from VisualDx helps you make more accurate diagnosis?	18 (82%)	0 (0%)	4 (18%)
Has VisualDx made your clinician work easier?	18 (82%)	0 (0%)	4 (18%)
Has VisualDx improved your ability to manage skin disease?	20 (91%)	0 (0%)	2 (9%)
Has VisualDx improved your ability to manage other (non-dermatologic) conditions?	14 (63%)	3 (14%)	5 (23%)
Would you benefit from further training on how to utilize VisualDx?	16 (73%)	6 (27%)	0 (0%)

**Table 3:** Rating of post-pilot participants’ experiences

Post Pilot Experiences (19 respondents)	Yes	No	Unsure
Have you encountered any issues or barriers when trying to use VisualDx?	11 (58%)	8 (42%)	0 (0%)
Have you encountered any scenarios where using VisualDx provided a clear benefit to you or your patient?	16 (84%)	3 (16%)	0 (0%)
Do you feel that the information you gain from VisualDx helps you make more accurate diagnosis?	17 (89%)	0 (0%)	2 (11%)
Has VisualDx made your clinician work easier?	17 (89%)	0 (0%)	2 (11%)
Has VisualDx improved your ability to manage skin disease?	18 (95%)	0 (0%)	1 (5%)
Has VisualDx improved your ability to manage other (non-dermatologic) conditions?	15 (79%)	1 (5%)	3 (16%)
Would you be interested in writing or reviewing medical content to be included in the VisualDx product?	13 (68%)	4 (21%)	2 (11%)

**Table 4:** Challenges categorized to themes and resolutions while implementing VisualDx at healthcare facilities in Botswana

Challenges or themes	Resolutions
Weak Internet connectivity	Purchase of mobile data bundles and training on how to utilize offline features.
Poor image quality	Demonstrations on good quality image capture for analysis by DermExpert feature.
Continuous health worker training	Use of pre-recorded VisualDx videos for different use cases. The authors also encouraged discussions on WhatsApp groups and raised questions to employ their curiosity of the capabilities of VisualDx.
Weak AI Regulation	Alignment with global AI ethical principles (for example, *Transparency, Impartiality, Accountability, Reliability, Security and Privacy*)
Sustainable funding for mHealth and CDSS	Engagement of the Ministry of Health and application to external funding opportunities
Participant retention	Continuous outreach to individual clinicians both within public and private sectors to replace churned participants. Offered free trial licenses to incentivize continued participation. Performed data analysis on each data collection instrument based on number of active participants instead of initial enrolled number.

The VisualDx features highlighted in participant training sessions included the ability search directly for a diagnosis to view expert-curated medical information and images (Fig. 1), the ability to build a differential diagnosis based on a patient’s symptoms to review diagnostic possibilities in situations of uncertainty (Fig. 2), and the ability to take a photo of a skin lesion with a mobile device and submit it to VisualDx’s DermExpert™ machine learning algorithm which identifies lesion morphology and diagnostic possibilities for dermatologic cases (Fig. 3).

Figure 4 illustrates the actual usage patterns of the participants. Building a custom differential diagnosis (Fig. 2) accounted for 48% of all usage, followed by 25% usage of direct diagnosis search (Fig. 1), and 22% usage of DermExpert™ (Fig. 3). The remaining 5% of usage was accounted for by other miscellaneous app features. Figure 4 also illustrates that most usage (65%) occurred using the VisualDx Android application, while 35% of usage occurred on the iOS application. A negligible amount of usage occurred on web browsers. Finally, 79% of usage occurred in online mode with internet access, while 21% utilized offline mode.

Experiences while using VisualDx were also noted and summarized. Overall, 22 (78.5%) study participants responded to the mid pilot survey while 19 (67.8%) responded to the post pilot study survey. During the mid-pilot survey, an average score of 4.36 was recorded by participants when responding to the quality and relevance of the VisualDx diagnosis content (1 = irrelevant and 5 = relevant). An average score of 4.36 (1 = irrelevant and 5 = relevant), was also recorded for the same question post the study pilot phase. On another note, when asked to rate how easy it was to use VisualDx (1 = Difficult and 5 = Easy), an average score of 4.27 was recorded during the mid-pilot study phase while 4.42 recorded during the post-pilot phase. Study participants across mid and post pilot phases reported their top three useful features of VisualDx to be, (1) creating differential diagnosis, (2) looking up diagnosis and (3) looking at photos of various skin conditions.

Tables 2 and 3 summarize ratings of participants’ experiences during the mid-pilot and post-pilot phases respectively.

Challenges and resolutions while implementing the VisualDx platform to support healthcare facilities in Botswana are summarized (Table 4).

## DISCUSSION

This paper outlines the experiences, challenges and lessons learned during the implementation of the VisualDx platform in health facilities across Botswana. The challenges and lessons emerging from the study have been organized into five thematic categories: (*1) Infrastructure, (2) Capacity Development, (3) Artificial Intelligence (AI) Regulation, (4) Sustainable Health Technology Funding and (5) Participant Retention.*

### Infrastructure

Previous studies have highlighted the critical importance of robust ICT infrastructure for the successful and sustainable implementation of eHealth initiatives [21–24]. Mauco *et al*. [25] identify the presence of appropriate eHealth infrastructure as a fundamental determinant of a setting’s readiness to adopt eHealth solutions. In line with this, the World Health Organization (WHO) has reported several barriers to eHealth adoption across Africa, including limited ICT budgets, inadequate communication infrastructure to support health services and inconsistent electricity supply—all of which are indicative of poor eHealth readiness [26].

Recognizing these challenges, the Botswana National eHealth Strategy prioritizes the development of foundational infrastructure as a key enabler of eHealth. Specific infrastructure-related objectives targeted for completion by 2023 include: (1) connecting all health facilities in the country with a minimum required bandwidth, (2) establishing registries and national data dictionaries and (3) implementing a Master Patient Index (MPI) to support continuity of care across all levels of the healthcare system [6]. The strategy also acknowledges the value of open-source software solutions endorsed by the WHO and the necessity of a centralized national data repository (data warehouse) to support integrated health information management [6].

In this study, one of the key infrastructure challenges encountered was the slow internet speed at public health facilities connected to the Government Data Network (GDN). The GDN is a shared resource that provides internet connectivity to various government ministries and departments, which may have contributed to bandwidth limitations and reduced network performance during the implementation of VisualDx in these facilities.

To address the challenge of slow internet connectivity at public health facilities, the authors partnered with private internet service providers (ISPs) to deliver internet access via mobile routers. This mitigation strategy aligns with the recommendations of the Botswana National eHealth Strategy, which advocates for the involvement of private sector stakeholders in supporting eHealth implementation, particularly under the ‘Strategy and Investment’ pillar [6]. In contrast, private health facilities did not experience connectivity issues, as they were already equipped with independently sourced internet services.

An additional mitigation strategy for addressing internet connectivity challenges was the deployment of an offline-capable version of VisualDx for participants using Android devices. This feature served as a backup option, enabling users to access the CDSS tools even during periods of slow or intermittent connectivity. The offline mode was particularly suited to devices with limited storage capacity or low power performance, such as smartphones, as previously highlighted in the literature [27]. However, in this study, most participants opted to use VisualDx in online mode (Fig. 4). This preference was likely influenced by the availability of privately procured internet data bundles provided for the study, as well as participants’ concerns about memory constraints on their personal mobile devices.

### Capacity development

Consistent with findings from other studies in low- and middle-income countries, there is a recognized shortage of trained personnel to support the implementation and maintenance of Clinical Decision Support Systems (CDSS) [28–30]. However, this challenge is not exclusive to the developing world; it is also evident in high-income countries. Initiatives such as the American Medical Informatics Association’s 10x10 education program, the Informatics Training for Global Health Program supported by the Fogarty International Center, and the integration of medical informatics courses into university curricula represent efforts to address these workforce gaps [31].

In Botswana, the National eHealth Strategy (2020–2024) identifies strengthening the Ministry of Health’s human resource capacity as a key priority. Specifically, it emphasizes the need to enhance the ability to generate, disseminate, access, secure, store and use health information for evidence-based planning and policymaking at all levels (subsection 1.3.2) [6]. This aligns with the WHO Global Strategy on Digital Health [2], which underscores the critical importance of capacity building and digital literacy among healthcare workers. According to the strategy, effective digital health systems require high-quality data collection and the capacity to share this data to inform planning, service delivery and health system transformation. Furthermore, the strategy highlights the need for targeted investments in institutional and workforce development, asserting that digital health can significantly improve health outcomes if implemented alongside national strategies.

As healthcare institutions expand their digital infrastructure, there is also a growing need to enhance awareness and understanding of digital privacy laws and regulations among healthcare professionals [32].

In this study, VisualDx contributed to the empowerment of healthcare workers by supporting more accurate diagnoses (Table 2) and enhancing their ability to manage both dermatologic and non-dermatologic conditions (Table 3). Additionally, a modest improvement in perceived ease of use was observed between the mid-pilot and post-pilot phases (Tables 2 and 3), which may be attributed to the effects of targeted training and increased familiarity with the platform over time. As a result of this growing confidence and competence, a majority of participants (68%) expressed interest in contributing to the development of the VisualDx platform by authoring or reviewing medical content (Table 3).

### AI ethics and regulation

In recent years, there has been a growing interest in the application of AI in healthcare, with the aim of creating intelligent processes and workflows that can enhance the affordability, effectiveness, personalization and equity of care delivery [33]. However, the World Health Organization (WHO) urges caution in the deployment of AI tools—particularly large language models (LLMs)—to safeguard human well-being, safety and autonomy and to preserve public trust in health systems [34]. The WHO reinforces the foundational medical principle of primum non nocere (‘do no harm’) [35], asserting that in the digital age, this principle must also apply to AI-driven technologies. Properly implemented, AI has the potential to reveal clinical best practices by analyzing patterns in electronic health records (EHRs), thereby informing innovations in healthcare delivery, such as precision medicine [36].

Despite this promise, regulatory frameworks for AI in healthcare remain underdeveloped in many low- and middle-income countries, including Botswana. There is a pressing need for the establishment of standards for ‘good machine learning practices’ and for robust oversight mechanisms to ensure safe, ethical and effective integration of AI technologies into healthcare systems.

A recent study identified that in order ‘to fully achieve the potential of AI in healthcare, four major ethical issues must be addressed: ***(1) informed consent to use data, (2) safety and transparency, (3) algorithmic fairness and biases or discrimination and (4) data privacy***’ [37]. Ethical considerations adhered to during implementation of VisualDx in Botswana entail;

Obtaining informed consent from all participants, maintaining data privacy and confidentiality, providing clear information on the functioning and limitations of the platform to ensure transparency and promoting equitable access and use of the tool across different healthcare settings to mitigate potential bias or discrimination.

VisualDx utilizes peer-reviewed and expert-validated clinical content and is compliant with both the General Data Protection Regulation (GDPR) [38] and the Health Insurance Portability and Accountability Act (HIPAA) [39], ensuring adherence to international standards for data protection and patient privacy.

To promote algorithmic fairness and minimize biases, the DermExpert feature within VisualDx leverages convolutional neural networks (CNNs) [40], a widely used deep learning architecture for computer vision tasks. The CNN model was trained on over 80 million image variations representing a broad spectrum of ethnicities and skin tones, including both dark and light skin. Notably, the model was extensively tested on dark-skinned individuals in Botswana, contributing to a federated learning approach [41] that supports continuous improvement and fairness of the algorithm across diverse populations.

Lastly, in adherence to data privacy principles, VisualDx collects only de-identified and generalized demographic information necessary to generate a differential diagnosis. When utilizing the ‘DermExpert’ AI tool, patient images remain stored locally on the user’s device and are immediately discarded after the analysis is completed. This design mitigates data security risks and ensures compliance with international data protection regulations, including the Health Insurance Portability and Accountability Act (HIPAA) [39] and the General Data Protection Regulation (GDPR) [38].

### Sustainable health technology funding

While results from the study indicated promising acceptance of the VisualDx platform by study participants, in the months since the primary data collection period, the authors have been challenged to overcome what is commonly referred to as ‘pilotitis,’ or the tendency to stall and remain in the pilot phase. Although VisualDx had initial funding for a pilot study, it was not sufficient to support its implementation at national level. This challenge has been highlighted by others in the literature, with reference to the need for sustainable funding mechanisms to carry digital health implementations beyond the pilot phase [42]. Barriers such as lack of regulatory frameworks and patients’ data ownership issues have been highlighted to also contribute to ‘pilotitis’ [43].

Sustainable funding is widely recognized as a critical enabler for scaling up digital health interventions [43]. Egermark *et al*. propose several potential funding models, including (1) insurance-based healthcare financing systems, (2) reimbursement mechanisms for digital health technologies, (3) single-payer healthcare financing models and (4) dedicated funding for digital transformation—all of which could drive cost-effective improvements in care quality and patient outcomes [43]. This need for a sustainable digital health funding framework is also highlighted in the Botswana National eHealth Strategy (2020–2024), which calls for increased government budget allocations for eHealth, engagement of the private sector and donor agencies to support implementation and the establishment of an investment committee to facilitate resource mobilization across the healthcare system [6]. In alignment with the Strategy’s ‘Investment Pillar,’ the authors engaged the Ministry of Health and pursued external funding opportunities to support the implementation of VisualDx in Botswana.

### Participant retention

During implementation of VisualDx in Botswana, the authors experienced a challenge in maintaining a high participants’ retention rate as evidenced by findings in Tables 2 and 3 where the latter showed an increase in the number of participants who experienced barriers in using the platform. This was partly attributable to the unpredictable nature of participants’ work conditions during the COVID-19 pandemic. To mitigate this challenge, the authors provided free VisualDx trial licenses as an incentive for continued participation and conducted data analysis for each collection instrument based on the number of active participants, rather than the initially enrolled cohort (Table 2). Providing incentives and nudges was also previously reported as having the potential to improve study participation rate [44].

It is worth noting that the response rates from each VisualDx survey dropped off as the study went on, that is, the pre-pilot survey had 100%, mid-pilot survey was completed by 78.6% participants and the post-pilot survey had 67.8% rate. The survey length was not considered a contributing factor as the pre-pilot survey had 29 questions, mid-pilot survey had 22 questions and the post-pilot survey had 19 questions. This however, could have been that participants’ got fatigued along the way as the survey activities were conducted far apart. A similar observation was previously noted in a study by Daniore *et al*., where infrequent tasks with higher cognitive burden contributed to minimal participation [44].

## STUDY LIMITATIONS

The study protocol was impacted by restrictions and delays associated with the COVID-19 pandemic. As a result, training and interview sessions were conducted remotely using the Zoom platform. Additionally, researchers were unable to visit clinics in person to provide technical support or conduct on-site training.

Overall, participation in the study was limited, in part due to the reassignment of some healthcare workers to COVID-19-related duties such as vaccine distribution, which did not involve the use of VisualDx. Additionally, increased stress levels and heavy workloads associated with COVID-19 surges in Botswana likely contributed to reduced compliance in using the VisualDx platform.

## CONCLUSION

The implementation of VisualDx in Botswana illustrates both the value and the challenges of cross-sector and cross-border collaboration in driving adoption of eHealth tools in developing healthcare systems. Understanding and preparing for these challenges may inform and improve future strategy for implementation and research of other eHealth platforms in Botswana and other countries with similar levels of infrastructure readiness, capacity development, funding and AI regulation.

## Data Availability

Data supporting the findings of this study are available from the corresponding author upon reasonable request.
